# Regurgitação Mitral após Infarto Agudo do Miocárdio: Uma Condição Multifacetada

**DOI:** 10.36660/abc.20240707

**Published:** 2025-01-30

**Authors:** Carlos Henrique Miranda

**Affiliations:** 1 Universidade de São Paulo Faculdade de Medicina de Ribeirão Preto Departamento de Clínica Médica Ribeirão Preto SP Brasil Divisão de Medicina de Emergência, Departamento de Clínica Médica, Faculdade de Medicina de Ribeirão Preto, Universidade de São Paulo, Ribeirão Preto, SP – Brasil

**Keywords:** Insuficiência da Valva Mitral, Infarto do Miocárdio, Revascularização Miocárdica

Existem duas causas principais de regurgitação mitral (RM) após infarto agudo do miocárdio (IAM). Primeiro, a ruptura parcial ou completa do músculo papilar leva à RM; neste caso, o reparo cirúrgico é obrigatório. Felizmente, a prevalência desta condição é baixa, em torno de 0,05%.^1^ Segundo, a válvula e o aparelho subvalvar são normais, e a RM resulta da amarração do folheto e da dilatação regional do ventrículo esquerdo. O mecanismo primário é a separação e a angulação excessiva do músculo papilar relacionada ao movimento regional da parede na área do infarto do miocárdio. Essa segunda apresentação é chamada de fenótipo "funcional".^2^ Esta RM funcional é um achado ecocardiográfico comum após IAM. Uma investigação recente mostrou uma RM leve em 76%, RM moderada em 21% e RM grave em 3% dos pacientes após IAM.^3^ Vyas et al.^4^ encontraram uma prevalência significativa de RM (moderada + grave) de 7,21% após infarto do miocárdio sem supradesnivelamento do segmento ST (IAMSSST). Um aspecto importante a destacar é que a maioria dos estudos anteriores analisou a prevalência de RM em todos os tipos de infarto do miocárdio juntos ou IAM com supradesnivelamento do segmento ST sozinho.^5^ A prevalência real de RM no IAMSST era desconhecida, e este estudo mostrou uma prevalência considerável de RM neste cenário.

De acordo com outros estudos anteriores,^5,6^ esta investigação recente mostrou que a RM significativa piora o prognóstico dos pacientes, aumentando o risco de morte, edema pulmonar, insuficiência cardíaca e choque cardiogênico. Não há dúvidas sobre o impacto clínico da RM após IAM. O problema mais significativo é que a mortalidade associada a essa complicação não mudou por décadas, e ainda há muitas dúvidas sobre o melhor manejo desses pacientes.^2^ Outra característica merece ser comentada: as repercussões clínicas da RM grave são muito mais documentadas do que as da RM moderada, e essa classificação é crítica na tomada de decisões sobre o tratamento. Nesta investigação recente, os pesquisadores analisaram a RM moderada e grave juntas, chamadas de RM significativa; era compreensível devido ao pequeno tamanho da amostra de RM grave; no entanto, no cenário clínico, essas duas classificações têm desfechos totalmente diferentes durante o acompanhamento.

**Figura 1:**
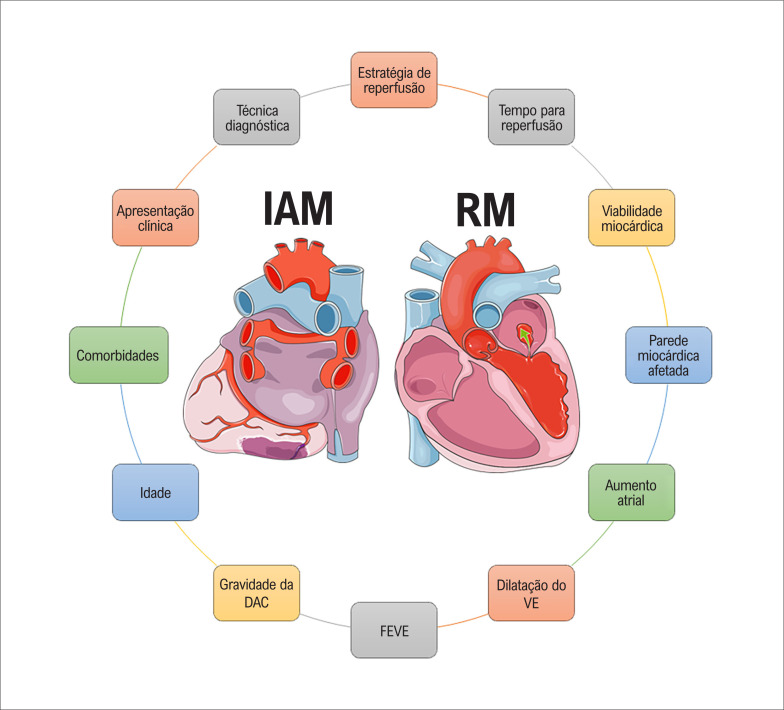
Ilustração das múltiplas características que podem interferir no prognóstico e na decisão sobre o tratamento em pacientes com insuficiência mitral (RM) após infarto agudo do miocárdio (IAM). DAC: doença arterial coronária; VE: ventrículo esquerdo; FEVE: fração de ejeção do ventrículo esquerdo

A RM após IAM é uma condição multifacetada. Diversas variáveis podem interferir no prognóstico e na decisão sobre o tratamento, por exemplo, a estratégia de reperfusão (intervenção coronária percutânea [ICP] versus cirurgia de revascularização do miocárdio [CRM]), o momento da reperfusão, a apresentação clínica (assintomática vs. edema pulmonar vs. choque cardiogênico), o território miocárdico regional afetado (parede inferior basal versus outro), a gravidade da doença arterial coronária (doença triarterial versus doença uniarterial), comorbidades (IAM prévio, diabetes), fibrilação atrial com aumento atrial, dilatação do ventrículo esquerdo (VE), fração de ejeção do ventrículo esquerdo (FEVE), a viabilidade miocárdica, a técnica diagnóstica empregada e várias outras.^2,5,6^Figura 1. Por isso, a análise multivariável é fundamental para avaliar os resultados desta pesquisa. Quando Vyas et al.^4^ analisaram a importância prognóstica da RM significativa, eles ajustaram por idade, diabetes, FEVE e doença multiarterial. A razão de chances diminuiu gradualmente à medida que novas variáveis foram incluídas no modelo. No entanto, ainda atingiu significância estatística, mostrando que a RM significativa foi um preditor independente de eventos. No entanto, esse modelo pode não ter incluído vários outros fatores de confusão.

Sobre o tratamento, a revascularização coronária imediata é a pedra angular na prevenção e tratamento de RM grave. Um estudo anterior mostrou que a revascularização miocárdica pode melhorar a RM em 32% dos pacientes.^2,5^ Vyas et al.^4^ exibiram uma melhora de 32,65% na RM com ICP, 6% com CRM e 16,98% com CRM + cirurgia de válvula mitral no acompanhamento de um ano, e essas intervenções melhoraram significativamente os resultados em comparação com o grupo de revascularização recusada e tratamento clínico. No entanto, esses três grupos são tão heterogêneos nas características basais que se tornou difícil estabelecer que toda essa melhora estava relacionada apenas à revascularização.

Atualmente, a maioria dos pacientes é tratada por meio de ICP após IAM. Nesse contexto, a substituição ou o reparo cirúrgico da valva mitral é mais complicado. O reparo transcateter edge-to-edge (TEER) para RM isquêmica aguda é um procedimento muito promissor. No primeiro estudo com 44 pacientes de alto risco cirúrgico, o sucesso técnico foi de 86,6%, e a mortalidade em 30 dias foi de 9,1%.^7^ Outra investigação avaliou o TEER em pacientes com RM e choque cardiogênico (53% com choque refratário, dois terços sob suporte de balão intra-aórtico ou Impella e 12% sob ECMO). O sucesso técnico foi alto e não diferiu entre pacientes com ou sem choque cardiogênico, e os eventos combinados (mortalidade/re-hospitalização) foram semelhantes entre pacientes com (28%) versus aqueles sem choque cardiogênico (25,6%), p=0,793.^8^ No entanto, a disponibilidade desse procedimento é restrita em todo o mundo, e os dados que o suportam ainda são limitados, mas estão em andamento. Essa opção de tratamento não foi considerada neste artigo.

Concluindo, a RM após IAM tem impacto clínico negativo mesmo no IAMSST aumentando o risco de morte, insuficiência cardíaca e choque cardiogênico. A revascularização é o primeiro passo no tratamento dessa condição porque pode melhorar a RM em um grande número de pacientes. A substituição ou reparo da valva mitral é indicada em pacientes com indicação de CRM e RM grave. No entanto, em pacientes tratados com ICP, a melhor opção e o melhor momento para intervenção na RM grave aguda são controversos.^9^ A RM após IAM é uma doença desafiadora, e vários aspectos devem ser considerados em conjunto para alcançar o melhor resultado para o paciente.
