# Supplementary material for: Regurgitação Mitral após Infarto Agudo do Miocárdio: Uma Condição Multifacetada
Source: Arq Bras Cardiol. 2025 Jan 30;121(12):e20240707. [Article in Portuguese] doi: 10.36660/abc.20240707 (PMC11809799; doi:10.36660/abc.20240707)
Supplement: Translation

# Translation: Mitral Regurgitation after Acute Myocardial Infarction: A Multi-Faceted Condition

**DOI:** 10.36660/abc.20240707i

**Authors:** Carlos Henrique Miranda

**Affiliations:** 1 Ribeirão Preto SP Brazil Divisão de Medicina de Emergência, Departamento de Clínica Médica, Faculdade de Medicina de Ribeirão Preto, Universidade de São Paulo, Ribeirão Preto, SP – Brazil

**Keywords:** Mitral Valve Insufficiency, Myocardial Infarction, Myocardial Revascularization

There are two leading causes of mitral regurgitation (MR) after acute myocardial infarction (AMI). First, the partial or complete papillary muscle rupture leads to MR; in this case, surgical repair is mandatory. Fortunately, the prevalence of this condition is low, around 0.05%.^1^ Second, the valve and the subvalvular apparatus are normal, and the MR results from leaflet tethering and regional left ventricular dilatation. The primary mechanism is the separation and excess angulation of the papillary muscle related to the regional wall motion in the myocardial infarction area. This second presentation is called the "functional" phenotype.^2^ This functional MR is a common echocardiographic finding after AMI. A recent investigation showed a mild MR in 76%, moderate MR in 21%, and severe MR in 3% of the patients after AMI.^3^ Vyas et al.^4^ found a significant MR (moderate + severe) prevalence of 7.21% after non-ST elevation myocardial infarction (NSTEMI). An important aspect to highlight is that most previous studies analyzed MR prevalence in all types of myocardial infarction together or ST-elevation AMI alone.^5^ The actual prevalence of MR in NSTEMI was unknown, and this study showed a considerable MR prevalence in this setting.

According to other previous studies,^5,6^ this recent investigation showed that significant MR worst the patients’ prognosis, increasing the risk of death, pulmonary edema, heart failure, and cardiogenic shock. There are no doubts about the clinical impact of the MR after AMI. The more significant problem is that the mortality associated with this complication has not changed for decades, and there are still many doubts about the better management of these patients.^2^ Another feature deserves to be commented on: the clinical repercussions of severe MR are much more documented than those of moderate MR, and this classification is critical in making decisions about the treatment. In this recent investigation, the researchers analyzed the moderate and severe MR together, called significant MR; it was understandable because of the small sample size of severe MR; however, in the clinical setting, these two classifications have entirely different outcomes during the follow-up.

The MR after AMI is a multi-faceted condition. Several variables can interfere with the prognosis and in the decision about the treatment, for example, the reperfusion strategy (percutaneous coronary intervention [PCI] versus coronary artery bypass grafting [CABG]), the timing of the reperfusion, clinical presentation (asymptomatic vs. pulmonary edema vs. cardiogenic shock), regional myocardial territory affected (inferior basal wall versus other), severity of coronary artery disease (triple vessels disease versus single-vessel disease), comorbidities (prior AMI, diabetes), atrial fibrillation with atrial enlargement, left ventricle (LV) dilatation, left ventricular ejection fraction (LVEF), myocardial viability, the diagnostic technique employed and several other.^2,5,6^Figure 1 Because of this, multivariable analysis is fundamental to evaluating the results of this research. When Vyas et al.^4^ analyzed the prognostic importance of the significant MR, they adjusted by age, diabetes, LVEF, and multivessel disease. The odds ratio decreased gradually as new variables were included in the model. However, it still achieved statistical significance, showing that significant MR was an independent predictor of outcomes. Nevertheless, this model could not have included several other cofounders.

**Figure 1:** Illustration of the multiple features that can interfere with the prognosis and the decision about the treatment in patients with mitral regurgitation (MR) after acute myocardial infarction (AMI). CAD: coronary artery disease; LV: left ventricle; LVEF: left ventricle ejection fraction

About the treatment, immediate coronary revascularization is the cornerstone in preventing and treating severe MR. A previous study showed that myocardial revascularization can improve MR in 32% of patients.^2,5^ Vyas et al.^4^ exhibited a 32.65% MR improvement with PCI, 6% with CABG, and 16.98% with CABG + mitral valve surgery at one-year follow-up, and these interventions significantly improved outcomes compared to refused revascularization and medical management group. However, these three groups are so heterogeneous at the baseline characteristics that it became difficult to establish that all this improvement was related to revascularization alone.

Nowadays, most patients are treated through PCI after AMI. In this context, surgical mitral valve replacement or repair is more complicated. The transcatheter edge-to-edge repair (TEER) for acute ischemic MR is a very promising procedure. In the first study with 44 high surgical-risk patients, the technical success was 86.6%, and the mortality at 30 days was 9.1%.^7^ Another investigation evaluated the TEER in patients with MR and cardiogenic shock (53% with refractory shock, two-thirds under support with an intra-aortic balloon pump or Impella, and 12% under ECMO). Technical success was high and did not differ between patients with or without cardiogenic shock, and the combined events (mortality /rehospitalization) were similar between patients with (28%) versus those without cardiogenic shock (25.6%), p=0.793.^8^ However, this procedure's availability is restricted worldwide, and data supporting it is still limited but in progress. This treatment option was not considered in this paper.

In conclusion, MR after AMI has a negative clinical impact even in NSTEMI, increasing the risk of death, heart failure, and cardiogenic shock. Revascularization is the first step in the treatment of this condition because it can improve MR in a large number of patients. Mitral valve replacement or repair is indicated in patients with CABG indication and severe MR. However, in patients treated with PCI, the best option and time for intervention in acute severe MR is controversial.^9^ The MR after AMI management is a challenging illness, and several aspects should be considered together to accomplish the best outcome for the patient.
